# Effects of surfactant head group modification on vertically oriented mesoporous silica produced by the electrochemically assisted surfactant assembly method†

**DOI:** 10.1039/d3na00031a

**Published:** 2023-05-17

**Authors:** Nabil A. N. Mohamed, Yisong Han, Sarah Harcourt-Vernon, Andrew L. Hector, Anthony R. Houghton, Gillian Reid, Daryl R. Williams, Wenjian Zhang

**Affiliations:** a School of Chemistry, University of Southampton Highfield Southampton SO17 1BJ UK A.L.Hector@soton.ac.uk; b Department of Physics, University of Warwick Coventry CV4 7AL UK; c Department of Chemical Engineering, Imperial College London SW7 2AZ UK

## Abstract

Production of mesoporous silica films with vertically oriented pores has been a challenge since interest in such systems developed in the 1990s. Vertical orientation can be achieved by the electrochemically assisted surfactant assembly (EASA) method using cationic surfactants such as cetyltrimethylammonium bromide (C_16_TAB). The synthesis of porous silicas using a series of surfactants with increasing head sizes is described, from octadecyltrimethylammonium bromide (C_18_TAB) to octadecyltriethylammonium bromide (C_18_TEAB). These increase pore size, but the degree of hexagonal order in the vertically aligned pores reduces as the number of ethyl groups increases. Pore accessibility is also reduced with the larger head groups.

## Abstract 2

Electrochemically assisted surfactant assembly of vertically aligned porous silica films is described using a series of cationic surfactants with increasing head sizes of the form [C_18_R_3_N]Br, (C_18_ = octadecyl; R_3_ = Me_3_, Me_2_Et, MeEt_2_ or Et_3_).

## Graphical abstract

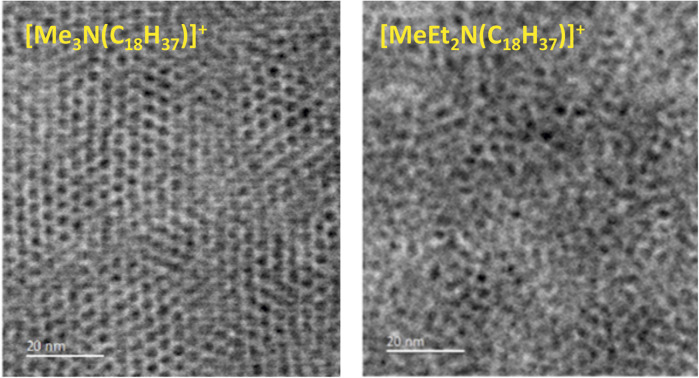

## Introduction

In the 1990s, Mobil researchers reported the M41S silica materials^1,2^ and since then mesoporous silicas have found many applications, including in catalysis,^3,4^ biosensing,^5^ electrochemical sensing^6^ and the encapsulation of metals, metal oxides and semiconductors to produce novel materials.^7–11^ Mesoporous silica materials possess attractive characteristic properties such as high specific surface areas, often greater than 1000 m^2^ g^−1^,^12^ well organised arrays of pores and pore size distributions in the range of 2–50 nm, depending on the method applied.

The most common approach to the synthesis of mesoporous silica films is by the evaporation induced self-assembly method (EISA).^15^ This process involves the use of tetraethylorthosilicate and a surfactant in a prepared ethanol/water solution. The solution is sprayed, spin-coated or dip-coated onto the surface and, as the solvent evaporates from the substrate surface, the concentration of the surfactant increases locally, driving the growth of surfactant micelles *via* self-assembly. The surfactant is often removed either by Soxhlet extraction or calcination. Zhao *et al.*^16^ studied the influence of cationic surfactants such as cetyltriethylammonium bromide (C_16_TEAB) and Gemini surfactants (C_18-3-1_) in the formation of mesoporous silica films by dip-coating from aqueous and non-aqueous solutions. It was demonstrated that silica films containing C_16_TEAB tend to produce 3D cubic pore structures in aqueous conditions, whereas under highly acidic conditions, 2D-hexagonal pores are favoured using the same surfactant. Under aqueous and non-aqueous environments, silica films with Gemini surfactant, C_18-3-1_, produce either 3D-hexagonal or 2D-hexagonal structures, where the pores in the 2D structures are oriented parallel to the surface.

Vertical pore orientation is rather more difficult to achieve in comparison to horizontal orientation using the EISA method. Vertically-oriented mesopores can be achieved by a Stöber process with C_16_TAB under basic conditions, but these methods suffer from long preparation times to make very thin films.^17^ An alternative method is electrochemically assisted surfactant assembly (EASA) using a cationic surfactant, usually C_16_TAB, and a silica precursor.^12,18^ A conductive substrate is placed in an ethanol/water sol containing a cationic surfactant and a negative potential is applied, resulting in the production of hydroxide ions that increase pH in the vicinity of the electrode, which accelerates the rate of Si–O–Si bond formation around the formed hemi-micelles. The surfactant is removed from the mesopores by washing with an acidic alcoholic solution or calcination for 30 minutes to generate mesoporous silica films with vertical pore channels. We previously reported variations in those oriented mesoporous silica films that can be achieved with C_*n*_TAB surfactants from C_14_ to C_24_.^19^ Faster ion diffusion rates were observed from a variety of redox probe molecules for films with increasing surfactant chain lengths, linked to increases in pore size. EASA most commonly uses C_16_TAB, resulting in small diameter pores of 2–3 nm diameter. Ryoo *et al.*^14^ showed that under hydrothermal conditions that would produce MCM-41 powders with CTAB, C_22_TAB produced lamellar structures. Post-synthesis grafting may facilitate better electrolyte access to the substrate surface during electrodeposition.^21^ The use of swelling agents such as mesitylene is also an alternative way to produce larger pores.^20,22^

The effects of various modifications to surfactants in the hydrothermal synthesis of mesoporous powdered silicas have been reported. Lin *et al.*^13^ investigated a surfactant chain length of 16 carbon atoms with various other head groups. The substitution of a methyl for an ethyl or benzyl group in the conventional C_16_TAB surfactant brought about a decline in pore order and a shrinkage in pore size from 4.0 nm (C_16_TAB) to 3.6 nm (cetyldimethylethylammonium bromide) surfactants. Ryoo *et al.*^14^ found that mixing C_*n*_TAB and alkyltriethylammonium bromide surfactants, with a carbon chain length from C_12_ to C_22_, could increase the structural order of MCM-41. Furthermore, Campos *et al.*^23^ produced aqueous dispersions of mesoporous silica using mixtures of C_16_TAB and cetyltripropylammonium bromide (C_16_TPAB) under hydrothermal conditions, showing that increasing the surfactant head group resulted in larger lattice spacings but also a significant reduction in hexagonal ordering. The understanding of such head group modifications in the EASA process, which works very differently due to surfactant ordering by the electric field, is limited to a study by Robertson *et al.*^20^ who evaluated the influence on the lattice parameters and pore ordering of switching the head group of the surfactant to a bulkier cetylpyridinium bromide (CPyB). The film became less well-organised, but an increase in the lattice spacings was noticed. In this paper, the porosity, structural pore order and pore size are systematically evaluated for porous silicas produced using the EASA method with cationic surfactants with a tetraalkylammonium head group containing a hydrophobic chain of 18 carbons, and a mixture of zero, one, two or three ethyl groups, with methyl groups making up the remainder, [C_18_H_37_NMe_3−*x*_Et_*x*_]Br with *x* = 0, 1, 2 or 3.

## Experimental

**Scheme 1:**
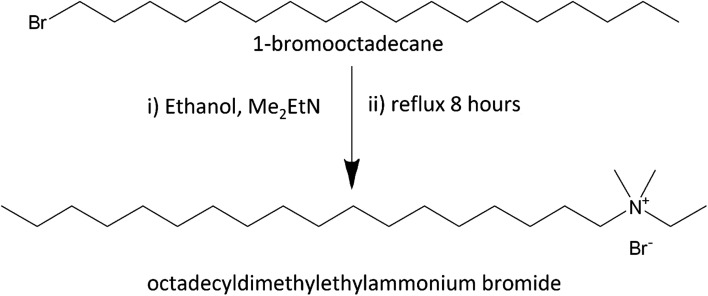
The reaction used to produce octadecyldimethylethylammonium bromide surfactant, C_18_DMEAB. The same method was used for the C_18_DEMAB and C_18_TEAB surfactants.

Octadecyltrimethylammonium bromide (C_18_TAB, 98%) was purchased from Sigma Aldrich. The other octadecylalkylammonium bromide surfactants were prepared by the reaction of the alkylamine and 1-bromooctadecane in ethanol according to Scheraga *et al.* (Scheme 1).^24^ The synthesis used 1-bromooctadecane (5.0001 g, ≥97%, Sigma Aldrich) which was dissolved in ethanol (25 cm^3^) and dimethylethylamine (10× mol eq., ≥99%, Sigma Aldrich), diethylmethylamine (10× mol eq., 97%, Sigma Aldrich) or triethylamine (10× mol eq., ≥99.5%, Sigma Aldrich) was added into the solution. The mixture was refluxed for 8 h under stirring at 100 °C using a dry ice condenser. The solution was filtered to remove particulates, then the filtrate was placed in the freezer. The crude product was collected by filtration and a rotary evaporator was used to reduce the ethanol solvent to recover the solid from the filtrate. Recrystallisation of the combined 2 crop crystals from ethanol produced a final yield of 4.98 g octadecyldimethylethylammonium bromide, (C_18_DMEAB, yield = 99% after drying, white solid), 4.80 g octadecyldiethylmethylammonium bromide (C_18_DEMAB, yield = 96% after drying, off-white solid) and 4.76 g octadecyltriethylammonium bromide (C_18_TEAB, yield = 95% after drying, off-white solid). ^1^H and ^13^C{^1^H} NMR and positive ion ESI MS data are shown in the ESI.†

All ^1^H and ^13^C{^1^H} NMR spectra for surfactants C_18_DMEAB and C_18_TEAB in D-chloroform and C_18_DEMAB in D4-methanol were run on a Bruker AV11-400 spectrometer at 25 °C. Solutions used for mass spectrometry analysis were prepared by dissolving 50 μg of surfactant in 1 mL of methanol. Ultrahigh performance liquid chromatography (UHPLC) coupled to a TQD mass tandem quadrupole mass spectrometer (Waters) with a TUV detector at 254 nm was used to analyse the surfactants. A Waters BEH C18 column (50 mm × 2.1 mm, 1.7 μm) with a flow rate of 0.6 mL min^−1^ was used for the chromatography. The mobile phase was 0.2% formic acid in an aqueous solution and 0.2% formic acid in acetonitrile. The mass spectrum in the positive ion electrospray ionisation mode was used for the data analysis of surfactants.

C_18_DMEAB: ^1^H NMR (CDCl_3_): *δ*/ppm 0.81 (t, CH_3_, [3H]), 1.17–1.39 (br s, CH_2_ and CH_3_, [33H]), 1.61–1.71 (m, CH_2_, [2H]), 3.32 (s, CH_3_, [6H]), 3.42–3.50 (m, CH_2_, [2H]), 3.66 (q, CH_2_, [2H]). ^13^C{^1^H} NMR (CDCl_3_): *δ*/ppm 8.57, 14.05, 22.61, 22.72, 26.24, 29.18, 29.28, 29.34, 29.41, 29.53, 29.58, 29.61, 29.63, 31.85, 32.78, 34.02, 50.64, 59.35, 63.46. MS (ESI^+^ in CH_3_OH): found *m*/*z* = 326.6; required for {C_22_H_48_N^+^}: *m*/*z* = 326.

C_18_DEMAB: ^1^H NMR (CDCl_3_): *δ*/ppm 0.81 (t, CH_3_, [3H]), 1.02–1.46 (br s, CH_2_ and CH_3_, [36H]), 1.61–1.75 (m, CH_2_, [2H]), 2.96 (s, CH_3_, [3H]), 3.14–3.25 (m, CH_2_, [2H]), 3.3 (q, CH_2_, [4H]). ^13^C{^1^H} NMR (CDCl_3_): *δ*/ppm 8.81, 14.09, 22.39, 22.66, 26.40, 28.15, 29.33, 29.39, 29.42, 29.45, 29.48, 29.52, 29.58, 29.63, 31.90, 32.83, 34.04, 56.62, 60.62. MS (ESI^+^ in CH_3_OH): found *m*/*z* = 340.6; required for {C_23_H_50_N^+^}: *m*/*z* = 340.

C_18_TEAB: ^1^H NMR (CDCl_3_): *δ*/ppm 0.81 (t, CH_3_, [3H]), 1.11–1.46 (br s, CH_2_ and CH_3_, [36H]), 1.29–1.41 (m, CH_3_, [3H]), 1.78 (m, CH_2_, [2H]), 3.28–3.48 (m, CH_2_, [8H]). ^13^C{^1^H} NMR (CDCl_3_): *δ*/ppm 8.15, 14.11, 22.13, 22.69, 26.55, 28.19, 28.77, 29.17, 29.36, 29.41, 29.44, 29.55, 29.59, 29.62, 29.66, 29.69, 31.93, 32.86, 34.02, 53.64, 57.65. MS (ESI^+^ in CH_3_OH): found *m*/*z* = 354.6; required for {C_24_H_52_N^+^}: *m*/*z* = 354.

### Growth of mesoporous silica on indium-tin oxide electrodes

The silica films were prepared on transparent indium-tin oxide (ITO) electrode using the EASA method previously reported by Goux *et al.*^25^ The sol electrolyte prepared from a 0.1 mol dm^−3^ sodium nitrate (0.1700 g, 2.00 mmol, >97%, Timstar Laboratory Suppliers Ltd) solution in 20 cm^3^ water and 20 cm^3^ ethanol, to which tetraethylorthosilicate (TEOS, 98%, Sigma Aldrich, 905 μL) and C_18_TAB (0.4801 g, 1.22 mmol) was added. The pH was adjusted to 3 using 0.2 mol dm^−3^ HCl in water and the sol was then hydrolysed for 2.5 h with stirring at 25 °C. For C_18_DMEAB (0.4801 g, 1.18 mmol), the ethanol component was replaced with 20 cm^3^ isopropyl alcohol to dissolve the surfactant at room temperature. When using C_18_DEMAB (0.4801 g, 1.14 mmol) and C_18_TEAB (0.4801 g, 1.10 mmol), in addition to using isopropyl alcohol it was necessary to increase the temperature to 40 °C to fully dissolve the surfactants.

The electrochemical cell for silica depositions consisted of a Teflon vessel with a 15 mm × 20 mm ITO-coated glass (surface resistivity = 14–16 Ω^−1^, Ossila), stainless steel cone counter electrode and a silver rod pseudo reference electrode. A constant potential of −1.25 V *vs.* Ag/Ag^+^ was applied for a duration of 20 s using a Biologic SP150 potentiostat. The silica films were quickly washed with water and ethanol after each deposition, then placed in a drying oven at 130 °C for 16 h. The surfactants were removed by washing the films in a solution of 0.2 mol dm^−3^ HCl/ethanol for 15 min.

### Film characterisation

Cyclic voltammetry (CV) was used to examine pore accessibility of a range of mesoporous silica films on transparent ITO electrodes using redox active molecules. For CV measurements, an aqueous solution of 0.5 mmol dm^−3^ potassium hexacyanoferrate(iii) ([Fe(CN)_6_]^3−^) and 0.1 mol dm^−3^ NaNO_3_ supporting electrolyte was used. The working electrode was a silica/ITO coated film, the counter electrode was a platinum gauze, and the reference was Ag/AgCl in 4 mol dm^−3^ KCl solution. Electrochemical impedance spectroscopy (EIS) data were recorded with a frequency range from 250 kHz to 100 mHz with a perturbation amplitude of 10 mV.

All grazing incidence small angle X-ray scattering (GISAXS) measurements were carried out using a Rigaku Smartlab X-ray diffractometer coupled with an incidence angle of 0.25° using a Hypix 2D detector and a copper K_α_ source. 1D patterns were recorded in-plane with 0.5° incident and 0.228° exit soller slits over a 2*θ* range of 1–10°. Scanning electron microscopy (SEM) images were recorded with a Jeol JSM-6500F and JSM-7200F with a 5 kV accelerating voltage. Scanning transmission electron microscopy (STEM) images of the mesoporous silica films were recorded using a JEOL ARM200F double-corrected TEM operated at 200 kV. TEM specimens were prepared by scraping the silica films off the substrate and suspending them on lacey carbon films. The TEM specimens were tilted during the TEM observations to allow for the establishment of an edge-on condition for some silica flakes (the pores directly facing the electron beam), when the size and the arrangements of the pores are clearly revealed.

Ellipsometric porosimetry (EP) experiments were performed using a dynamic vapour sorption (DVS) instrument (Surface Measurement Systems Ltd, UK) coupled with a FS-1 multi-wavelength ellipsometer (Film Sense, USA). The toluene vapour sorption experiments were carried out in an environmental chamber at a fixed temperature of 25 °C, at atmospheric pressure and a solvent partial pressure range between 0 and 95% *P*/*P*_0_. The partial pressure was maintained using mass-flow controllers operating in closed-loop mode using dry air as the carrier gas with a flow rate of 100 mL min^−1^ and the partial pressure monitored using a speed of sound sensor. The ellipsometer uses four wavelengths (465, 525, 595 and 635 nm) at an angle of incidence of approximately 65°, to produce the highest possible signal intensity in the detector. The mesoporous silica films were exposed to toluene in fixed partial pressure steps ranging between 0.5 and 5% for 20 min per step to reach equilibrium, and the refractive index was continuously measured to produce isotherms.

## Results and discussion

We investigated the impact of substituting one or more of the N-bound methyl groups on C_18_TAB for ethyl groups to determine whether increasing the head size would affect the pore growth/arrangements in EASA silica films and the electrochemistry of redox molecules in the presence of such films. Films were characterised with a range of analytical techniques including cyclic voltammetry, electrochemical impedance spectroscopy, GISAXS, electron microscopy and ellipsometric porosimetry.

### Pore structure and characterisation of EASA films

The surfactant chain length was kept constant at 18 carbons as the hydrophilic head was substituted with ethyl groups, thus steadily increasing the surfactant head size. The increase in head size also increased its hydrophobicity and reduced its solubility in the sol, so the sol composition was modified. The surfactant and TEOS concentrations were approximately 30 mmol dm^−3^ and 101 mmol dm^−3^ throughout, a surfactant : silica ratio of 0.3. As the solubility of the surfactant started to impede ordered porous silica deposition, the water : ethanol ratio was varied, *e.g.* for C_18_DMEAB 50 : 50, 75 : 25 and 15 : 85 mixtures were tried. When this approach failed, the water: ethanol mixture was replaced with water : isopropyl alcohol with a ratio of 50 : 50 and the desired ordered films were obtained. For C_18_DEMAB and C_18_TEAB, the 50 : 50 water: isopropyl alcohol-based sol required heating to 40 °C to achieve complete surfactant dissolution and hence EASA films with the preferred structure and orientation. Depositions were carried out at an applied potential of −1.2 V for 20 seconds. Longer deposition times resulted in a build-up of silica spheres on the ITO surface due to condensation reactions in the bulk sol.^18,19,25^ The sol electrolyte was maintained at approximately 40 °C during depositions for the surfactants with ethyl rich head groups, and the resulting films were then cleared of silica by-products using deionized water and ethanol on the surface and dried. Surfactant was removed from the pores by soaking in a solution containing ethanol acidified with a little HCl.

**Fig. 1:**
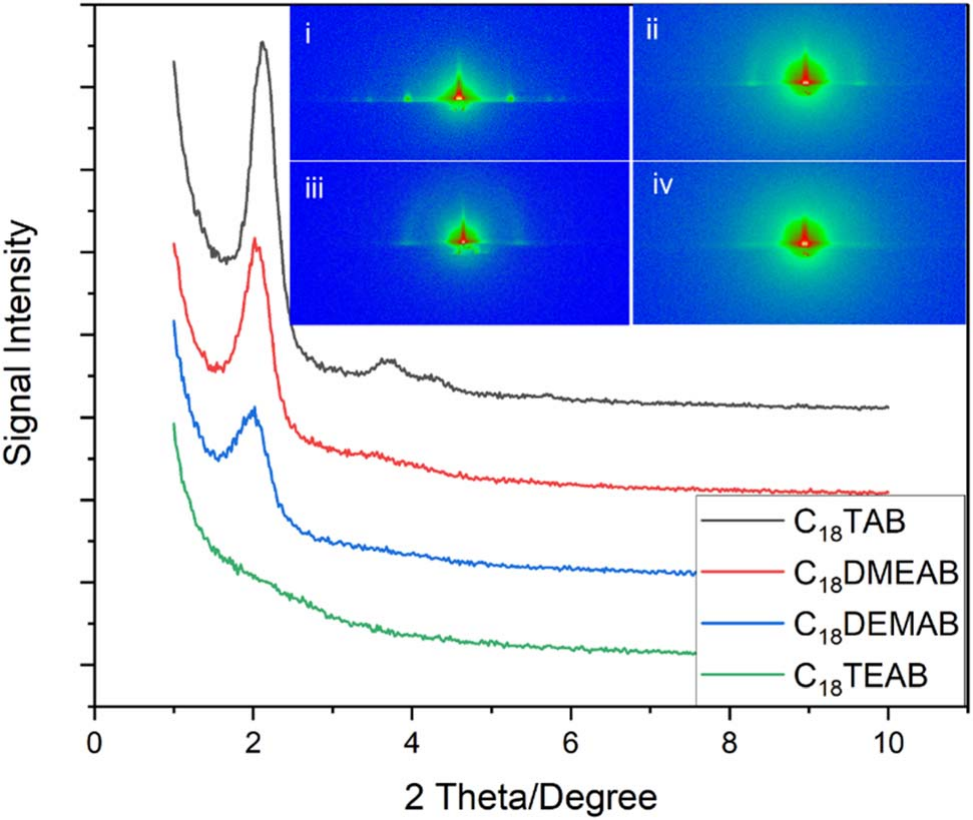
1D in-plane GISAXS patterns of EASA films produced with C_18_TAB, C_18_DMEAB, C_18_DEMAB and C_18_TEAB at a potential of −1.25 V (*vs.* Ag/Ag^+^) for 20 s on ITO electrodes (inset: 2D GISAXS images of EASA films templated by (i) C_18_TAB, (ii) C_18_DMEAB, (iii) C_18_DEMAB and (iv) C_18_TEAB).

In-plane GISAXS patterns (Fig. 1) were used to determine pore ordering and orientation in the silica films. All films were well-adhered, visually smooth, and free of any irregularities. The 1D in-plane scattering pattern for the silica film with C_18_TAB revealed 10 (2.13°), 11 (3.71°), 20 (4.35°) and 21 (5.71°) reflections of highly ordered mesoporous silica with hexagonal nanopores (space group *P*6*mm*). This is characteristic of the hexagonal patterns of MCM silica materials,^1,26^ C_*n*_TAB templated EASA films^19^ and hexagonal silica films produced by EISA.^27^ The 10 reflection position corresponds to a *d*-spacing of 4.15 nm and a pore spacing of *a*_H_ = 4.80 nm for the film templated by C_18_TAB. For the surfactant C_18_DMEAB, the displacement of a single methyl group with ethyl from the cationic head group saw a significant shift in the 10 (2.02°), 11 (3.50°) and 20 (4.18°) diffraction peaks. The 10 of the EASA film with C_18_DMEAB had a *d*-spacing of 4.40 nm resulting in a pore spacing of *a*_H_ = 5.10 nm. Campos *et al.*^23^ reported similar XRD features for the synthesis of mesoporous hybrid silica powders using cetyldimethylethylammonium bromide. In addition to increasing the pore spacing, modifying the surfactant head resulted in reduced intensity and broadening of the 10 diffraction peak, suggesting a reduction in hexagonal pore order for the silica film templated by C_18_DEMAB. The silica film containing C_18_TEAB gave rise to a disordered film, as indicated by the 1D GISAXS pattern shown in Fig. 1. A significant reduction in the peak intensity of the 10 diffraction peak and the absence of 11, 20 and 21 peaks were evident, suggesting a loss in the ordering of pores.

The 2D GISAXS in the inset of Fig. 1(i) clearly reveals well-defined diffraction spots in the horizontal plane corresponding to the 10, 11 and 20 reflections of the EASA film with C_18_TAB after removal of surfactant. The position of these spots demonstrates that the pores are aligned vertical to the plane of the substrate. Out of plane features or rings are not observed in the 2D pattern. Previous reports have linked the appearance of rings to the build-up of silica spheres on the electrode surface.^19,25^ The 2D diffraction images in the insets to Fig. 1(ii), (iii) and (iv) show EASA films templated by C_18_DMEAB, C_18_DEMAB and C_18_TEAB, respectively. These spots demonstrate that the films have pores oriented vertically to the surface. The 10 Bragg spots drop in intensity as the surfactant head size increases, and long range order also decreases, as evidenced by the loss of the higher order diffraction spots. Additional rings of low intensity are seen for the films with C_18_DMEAB and C_18_DEMAB, which become intense at longer deposition times. Such rings from EASA films with C_16_TAB, C_22_TAB and C_24_TAB have previously been attributed to the presence of silica spheres on the surface of the film after formation in the bulk solution.^19,25^

**Fig. 2:**
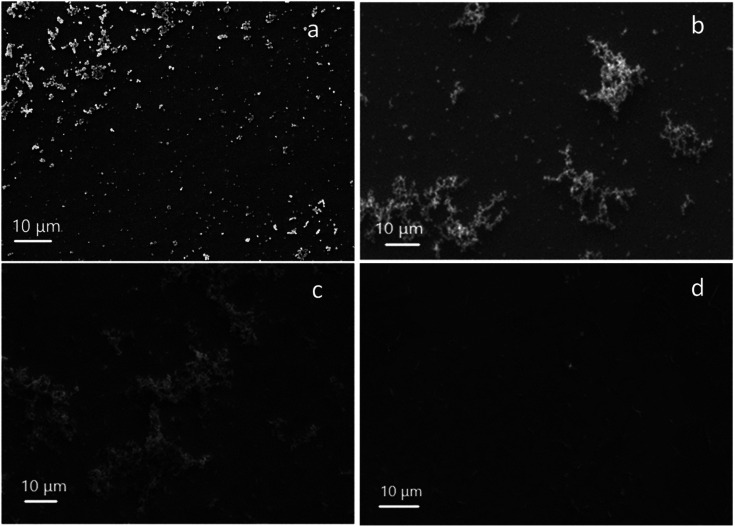
Top view SEM images of EASA films with (a) C_18_TAB, (b) C_18_DMEAB, (c) C_18_DEMAB and (d) C_18_TEAB deposited at an applied potential of −1.25 V (Ag/Ag^+^) for 20 s on ITO electrodes.

There are two ways to remove the surfactant template, either by washing in an acidic alcoholic bath or calcination. The former method was applied and the resulting porous silica films were characterised by SEM (Fig. 2). The top view SEM images of each film show good surface coverage with no signs of microcracks or poor adhesion. The estimated film thickness for the EASA film with C_18_TAB was 125 nm as determined by SEM and was found to be similar to the other EASA films. The silica aggregates noted above that result in diffraction rings are visible on the surface of the films.

**Fig. 3:**
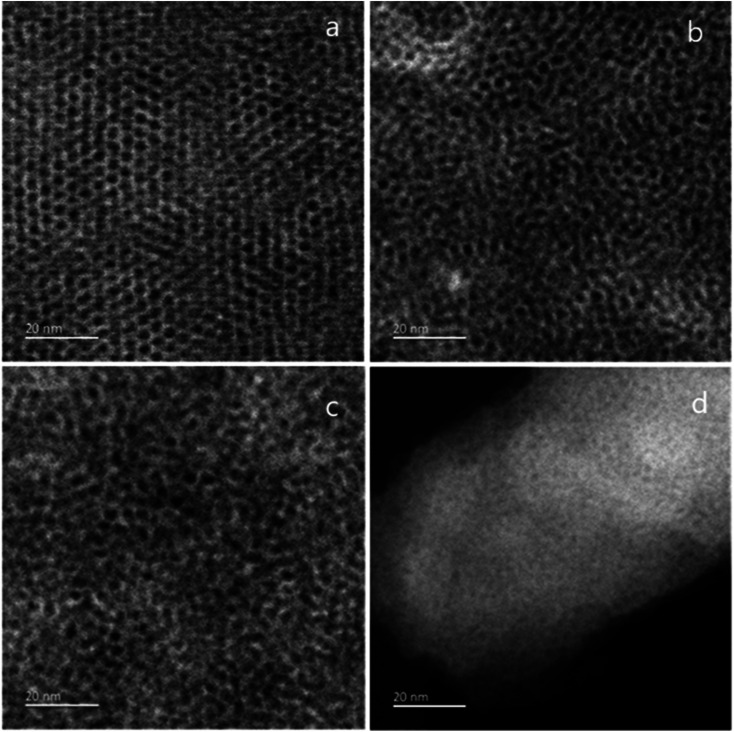
TEM images of mesoporous silica films generated with (a) C_18_TAB, (b) C_18_DMEAB, (c) C_18_DEMAB and (d) C_18_TEAB deposited at −1.25 V (*vs.* Ag/Ag^+^) for 20 s on ITO electrodes.

TEM images were taken by stripping small sections of film from the ITO surface to investigate the mesoporous structure. The GISAXS data (Fig. 1) had suggested a gradual loss of hexagonal organisation with increasing head group size. The TEM images in Fig. 3 show that the C_18_TAB produced film had a highly organised hexagonal structure of mesopores. As the surfactant head size increases, the hexagonally ordered domains become smaller with C_18_DMEAB. With C_18_DEMAB and C_18_TEAB, further loss in pore ordering is observed, which correlates with the GISAXS data in Fig. 1. It should be noted that the GISAXS confirms that all films possess vertically-oriented pores, it is only the hexagonal ordering that is lost in comparison to the film with C_18_TAB. Loss of order with increasing cationic surfactant head size has been linked to repulsions between the larger surfactant head groups causing steric hindrance to occur slowing down the process of micellization as a result of fewer bromide counterions condensing in the vicinity of the micelle.^13,28^ Film formation occurs below the critical micellar concentration (CMC) which is stated to be around 30 mmol dm^−3^ for CTAB in a sol electrolyte with 50 : 50 water : ethanol.^25,29^ This provides evidence that the surfactant template does not self-assemble in the desired hexagonal arrangement in the sol, it is the electric field which drives the interaction between positively charged surfactant molecule and the negatively charged ITO electrode. In turn, this favours the formation of hemimicelles (in its hexagonal shape) and induces silica condensation and hence SiO_2_ film formation with vertical pores. We are currently working below the CMC for this reason, so knowledge of the CMC is less critical here than it would be in a “typical” sol–gel study.

### Electrochemical characterisation of the films

Pore transit measurements were carried out for a range of EASA films using an anionic probe molecule, a 0.5 mmol dm^−3^ [Fe(CN)_6_]^3−/4−^ in a solution also containing 0.1 mol dm^−3^ NaNO_3_ as the background electrolyte. These investigations were undertaken to determine whether the effect of increasing the surfactant head size would influence the ability of ions to move through the pores. The electrochemical reduction and oxidation of ferricyanide is shown in eqn (1).1
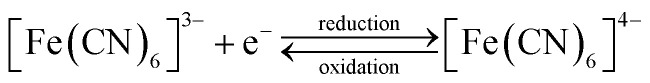


**Fig. 4:**
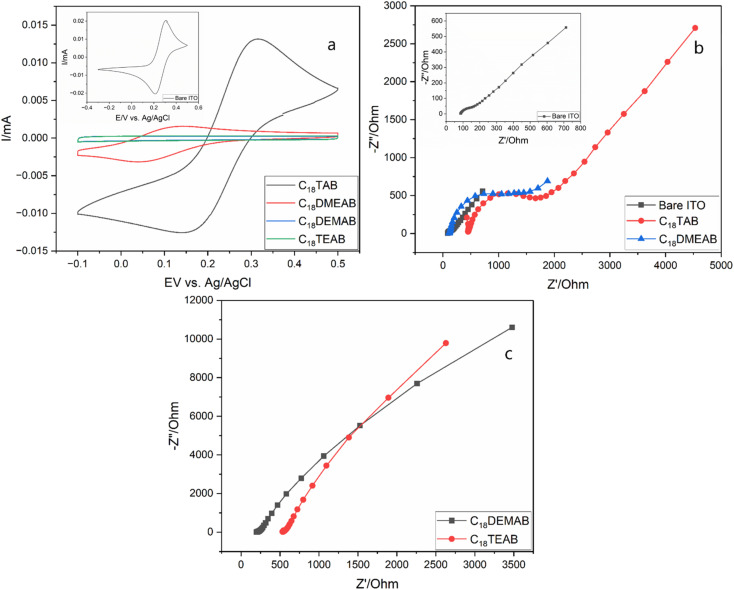
Cyclic voltammograms (a) (20 mV s^−1^ sweep rate) of 0.5 mmol dm^−3^ [Fe(CN)_6_]^3−/4−^ in 0.1 mol dm^−3^ NaNO_3(aq.)_ on bare ITO electrode (inset). The mesoporous silica films using C_18_TAB (black line), C_18_DMEAB (red line), C_18_DEMAB (blue line) and C_18_TEAB (green line) as the surfactants were deposited at −1.25 V (*vs.* Ag/Ag^+^) for 20 s on ITO electrodes. Nyquist plots in (b) are from bare ITO (inset) and mesoporous silica films on ITO electrodes with C_18_TAB and C_18_DMEAB as the surfactant templates, whereas Nyquist plots in (c) are from mesoporous silica films on ITO electrodes with C_18_DEMAB and C_18_TEAB as the surfactant templates. All experiments were carried out after surfactant removal.

Upon extracting the film's surfactant, a drop in peak current in the CVs and a large peak to peak separation is most likely due to electrostatic repulsions between the anionic molecule and silanol groups attached to the silica pore walls, which could be associated with the Donnan exclusion effect^30^ as seen for ionically charged regions in semi-permeable membranes (ESI, Table S1†).^31^ The peak current for the film produced with C_18_TAB (Fig. 4a) was similar to that of bare ITO (shown in Fig. 4a inset), but that with C_18_DMEAB was significantly lower. Films with C_18_DEMAB and C_18_TEAB reveal no faradaic current with the voltammograms adopting a rectangular shape, exhibiting capacitive behavior^32^ as well as resembling the films with blocked pores before removal of surfactant.^33,34^ GISAXS experiments were carried out before and after surfactant elimination to provide further evidence that the mesopores were indeed open for the film with C_18_DEMAB prior to CV studies (Fig. S10†). The featureless 1D GISAXS pattern indicates the presence of surfactant filled pores within the silica film however, after surfactant removal, a broad 10 reflection is noticed, suggesting that the surfactant has largely been removed from the pores. As the films' hexagonal pore order reduces due to the bulkier surfactant heads, a curtailment of the mass transport properties is found. This suggests the less ordered films have closed pores.

EIS was employed to further characterise the EASA films produced with the various head groups (Fig. 4b and c). The Nyquist plots were carried out in the same redox probe molecule solution as the pore transit measurements described above. The EIS plots can be simulated using a simple Randles equivalent circuit where *R*_s_, *R*_ct_ and *C*_dl_ are the electrolyte resistance, charge transfer resistance and double layer capacitance. The Nyquist plots of C_18_TAB and C_18_DMEAB produced silica films contain a semicircle in the high frequency region and a straight line with a 45° phase angle due to the Warburg impedance (*Z*_w_). Previous works have shown that mass transport of redox-active molecules through porous silica films is governed by diffusion processes.^12,19,25^ Therefore, the charge transfer resistance (*R*_ct_) is highly dependent on the number of open pores available for ion transport and ionic charge from redox active molecules. Fitting to the Randles circuit revealed a *R*_ct_ of bare ITO of 144.0 Ω, which was lower than the *R*_ct_ of 416.6 Ω and 830.2 Ω obtained in films produced by C_18_TAB and C_18_DMEAB, showing higher conductivity for the transparent electrode. The incorporation of two or three ethyl groups attached to the surfactant's hydrophobic tail resulted in much larger and imperfect semicircles (Fig. 4b and c), suggesting poorer pore accessibility of the redox active ion, [Fe(CN)_6_]^3−/4−^ transporting through the silica films than that seen with the C_18_TAB and C_18_DMEAB films. Moreover, despite the increased pore spacing and possible pore expansion found in C_18_DMEAB produced film, the *R*_ct_ value is higher than for C_18_TAB due to pore disorder and limited number of available pores present which hinder the diffusion of redox molecules within the silica pore channels. The EIS data agree with those from the CVs, presenting further evidence that the reducing pore order with increasing head size is accompanied by loss of access to the electrode surface.

### Ellipsometric porosimetry measurements of EASA films on ITO electrodes

**Fig. 5:**
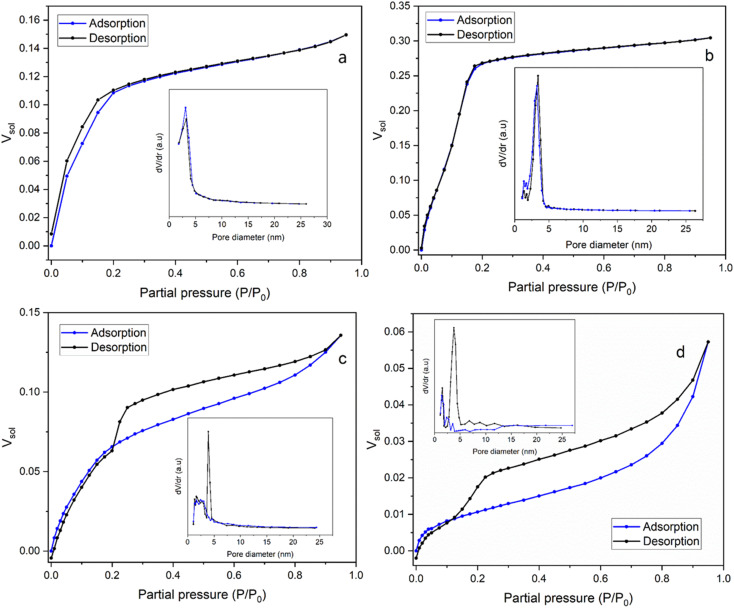
The adsorption/desorption isotherms of EASA films produced using (a) C_18_TAB (Mohamed *et al.*^19^), (b) C_18_DMEAB, (c) C_18_DEMAB and (d) C_18_TEAB surfactants using toluene as the adsorptive. The pore size distribution curves are shown in the insets.

Ellipsometric porosimetry (EP) is used to determine the porous structure of thin films deposited onto a solid substrate. Sorption isotherms, and hence pore size distribution and the open pore volume fraction, can be determined from the changes in refractive index of the film (Fig. 5). These changes are monitored by the film's exposure to toluene, which continuously condenses/adsorbs within the porous network. The high boiling point of toluene means that it easily condenses in the pores at relatively low partial pressure compared with highly polar solvents such as water.

In Fig. 5b the isotherm for the film templated with C_18_DMEAB shows a substantial initial uptake of toluene, with a minor step noticeable between 0.05 and 0.12 *P*/*P*_0_, and plateaus at around 0.175 *P*/*P*_0_. The adsorption/desorption process is also completely reversible with no significant hysteresis present, indicating the absence of any capillary condensation within the pores. The shape and characteristics of this isotherm are best described as a mix of type I (b) and type IV (b), with mixed monolayer-multilayer formation and micro-mesopore filling occurring up to the saturation.^35^ The plateau increases constantly until 0.95 *P*/*P*_0_, which does suggest some minor heterogeneity in the mesoporosity is present, as noticed in the pore size reduction after the peak in the pore size distribution (PSD). Both the adsorption and desorption branches of the PSD agree, with the latter branch having a peak pore size of around 3.42 nm (shown in Fig. 5b inset), which is larger than the peak pore size of the silica film with C_18_TAB at 3.24 nm (shown in Fig. 5a inset), determined from the desorption isotherm.^19^ The shape of this isotherm is common with templated porous materials with large micropores and small mesopores. Previous work on silica thin films templated by C_*n*_TAB^19,36^ has shown similar isotherms in the presence of toluene.

In the film templated by C_18_DEMAB (Fig. 5c), the isotherm initially shows broad adsorption of toluene from low partial pressures up to 0.2 *P*/*P*_0_, followed by a constant increase up to 0.8 *P*/*P*_0_ and ends with a minor inflection point. The desorption branch of the isotherm shows large hysteresis, implying that capillary condensation has occurred in the mesopores, and there is a significant drop in *V*_sol_ between 0.25 and 0.2 *P*/*P*_0_. The broad uptake of vapour at the lower end of partial pressures in the adsorption branch is a typical characteristic of overlapping monolayer formation and multilayer sorption occurring, in this case happening at a much more significant level than in the other films. This is a consequence of the film having a wide PSD range, rather than a sharp one, particularly within the larger micropore and narrow mesopore range. This can be seen in the adsorption branch of the PSD plot as a broad peak going up to 4–5 nm in addition to showing a gradual decrease in pore volume towards larger values. The shape of the isotherm best matches that of a mix between type I (b) and type II, with a type H4 hysteresis loop.^35^ The drop in *V*_sol_ in the desorption branch of this sample is a distinctive feature in this type of loop, which can be caused by the sample experiencing some degree of pore blockage or cavitation due to reduced porosity, pore size and pore geometry.^37^ This can happen, for example, when the larger pores in the structure have narrow entries or have neighbouring pores which are smaller in size, where the entry diameter generally dictates if pore blockage or cavitation will occur. A challenge with this phenomenon is that the sharp peak in the PSD of the desorption branch at 3.90 nm (Fig. 5c inset) could be an artifact due to the rapid desorption of the adsorbate because of the pore structure as opposed to the pore size, as described in a previous review.^38^ Therefore, for clarity, both the adsorption and desorption branches of the PSD are included in the plot, where they are in good agreement with each other except for the peak.

For the film templated by C_18_TEAB (Fig. 5d), the adsorption branch uptake shows clearer distinction between pore filling, monolayer coverage, and multilayer adsorption/condensation, like a type II isotherm. There is more uptake at higher partial pressure than lower ones, implying that there is more volume contribution from larger pores and voids. This is also seen in the PSD as there are multiple broad peaks present with low d*V*/d*r* at larger pore sizes. Although, the toluene volume fraction values are quite low for this sample, with a saturation *V*_sol_ of 5.73% at 95% *P*/*P*_0_, indicating low pore accessibility. The desorption branch shows significant hysteresis, with a rapid drop visible between 0.225 and 0.125 *P*/*P*_0_, and a shift towards lower partial pressures with a wider range. This is a similar phenomenon to C_18_DEMAB, that can cause an artificial peak in the desorption PSD, in this case at around 3.76 nm (Fig. 5d inset), which is larger than the peak pore sizes of 3.24 and 3.42 nm for C_18_TAB^19^ and C_18_DMEAB films (Fig. 5a and b insets). However, there is a smaller peak present in the adsorption branch at around 3.53 nm (Fig. 5d inset), which implies there is some distribution of pores at that size and not completely artificial. Even though the film has been prepared with a surfactant containing a larger head group, a shift to lower partial pressures in the drop range can imply a decreasing cavity diameter.^39^ At the same time, a more gradual drop in the range can indicate enlargement of necks/connected pores, which would mean that pore blockage is more likely occurring rather than cavitation.^40^ The shape and hysteresis of the isotherm resembles more of that with a type H3 hysteresis loop rather than type H4 for C_18_DEMAB, where there is less volume uptake at lower partial pressures due to lower micropore availability.^35^ The PSD shows quite some pore size heterogeneity, with other peaks also at smaller sizes visible in the adsorption branch, around 1.58 and 2.36 nm, although the BJH method is not very accurate at calculating the size of micropores. Both C_18_TEAB and C_18_DEMAB show some degree of hierarchical pore sizes and do not necessarily have very ordered structure. Isotherms of these shapes and pore blocking/cavitation phenomena have been associated with a range of ordered and disordered porous materials such as clays, sandstones, zeolites, and other silica-based materials.^37^

## Conclusions

A series of surfactants based on C_18_TAB with increasing numbers of ethyl groups replacing the usual methyl ones were used to synthesise vertically aligned mesoporous silica films by the electrochemically assisted self-assembly method. A gradual reduction in the pore order was observed as the surfactant head size grew, but alignment of the pores remained vertical. Introducing one ethyl group, an increase in the lattice spacing and pore size was observed without a significant reduction in hexagonal pore arrangement relative to the film produced with C_18_TAB. Resistance to transit of redox active species through the film did increase, however. No faradaic current was observed for films templated with surfactants with two or three ethyl groups, and pore order was also reduced (while again retaining vertical pore orientation). Ellipsometric porosimetry confirmed that the pore diameter of the silica films increased with increasing head group size, although accessible pore volume also decreased.

## Data availability

Raw data used to prepare the figures in the article and supplementary data are available from the University of Southampton repository at https://doi.org/10.5258/SOTON/D2638.

## Conflicts of interest

There are no conflicts to declare.

## Supplementary Material

NA-005-D3NA00031A-s001
