# Supplementary material for: Effects of surfactant head group modification on vertically oriented mesoporous silica produced by the electrochemically assisted surfactant assembly method
Source: Nanoscale Adv. 2023 May 17;5(12):3316–25. doi: 10.1039/d3na00031a (PMC10263000; doi:10.1039/d3na00031a)
Supplement: NA-005-D3NA00031A-s001 [file NA-005-D3NA00031A-s001.pdf]

Electronic Supplementary Material (ESI) for Nanoscale Advances.
This journal is © The Royal Society of Chemistry 2023

Supplementary information: Effects of surfactant head group modification on vertically oriented mesoporous silica produced by the electrochemically assisted surfactant assembly method

Nabil A. N. Mohamed,[a] Sarah Harcourt-Vernon,[b] Yisong Han,[b] Andrew L. Hector,[a]* Anthony R. Houghton,[c] Gillian Reid,[a] Daryl R. Williams[c] and Wenjian Zhang[a]

[a] School of Chemistry, University of Southampton, Highfield, Southampton SO17 1BJ, UK

[b] Department of Physics, University of Warwick, Coventry CV4 7AL, UK

[c] Department of Chemical Engineering, Imperial College London SW7 2AZ, UK

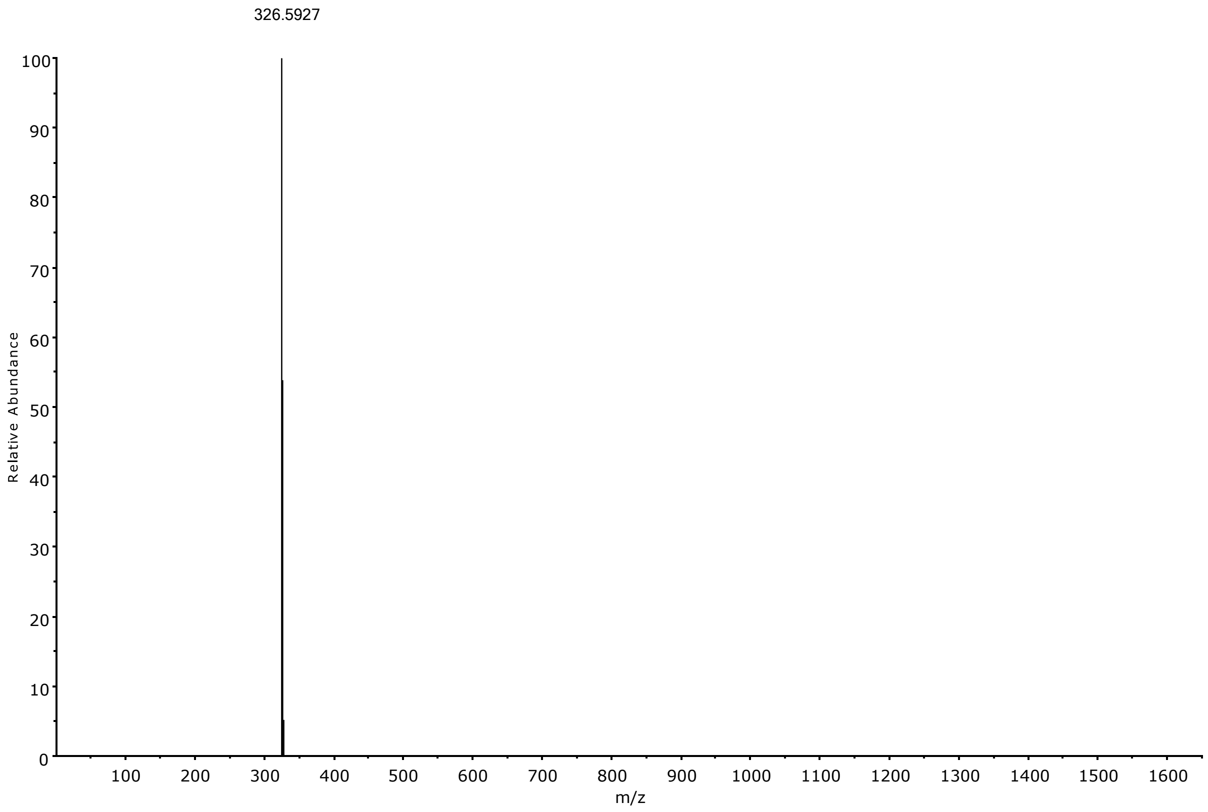

Figure S1. Positive ion electrospray mass spectrum of C_{18}DMEAB in methanol at 25 °C.

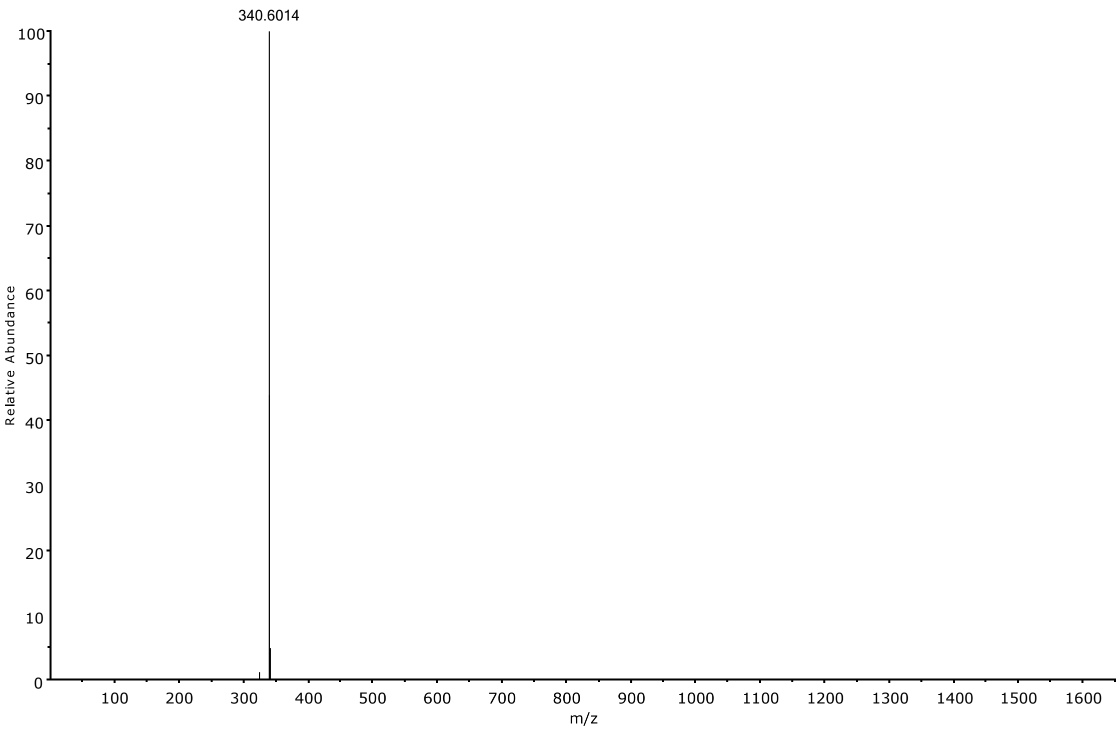

Figure S2. Positive ion electrospray mass spectrum of C_{18}DEMAB in methanol at 25 °C.

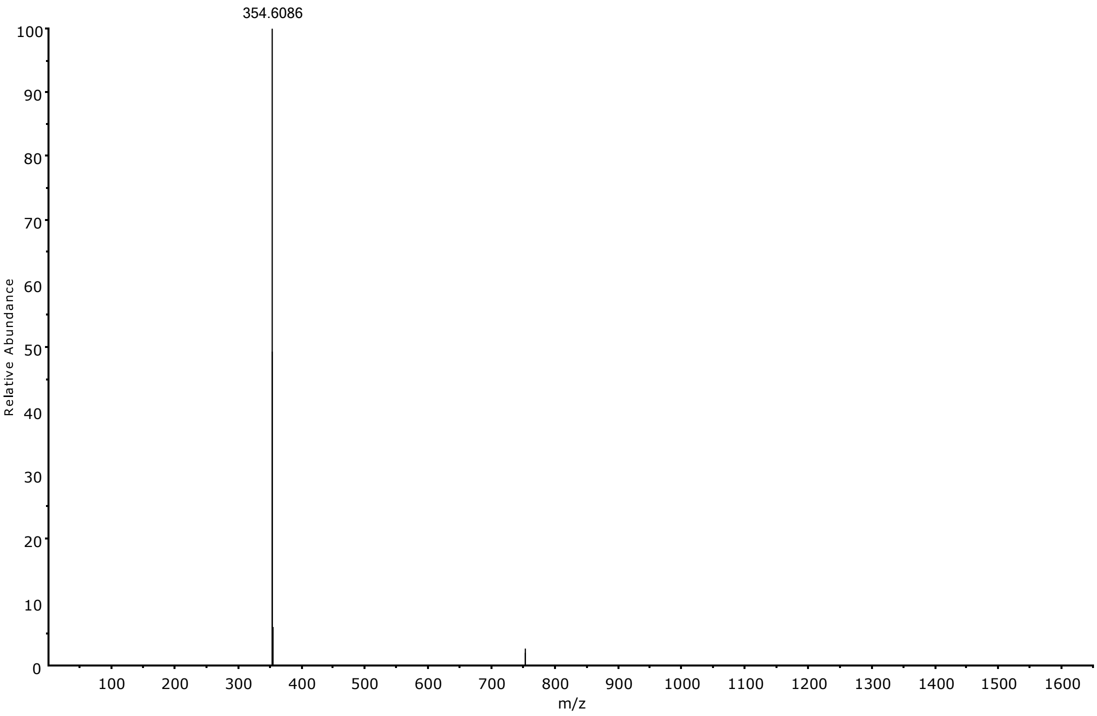

Figure S3. Positive ion electrospray mass spectrum of C_{18}TEAB in methanol at 25 °C.

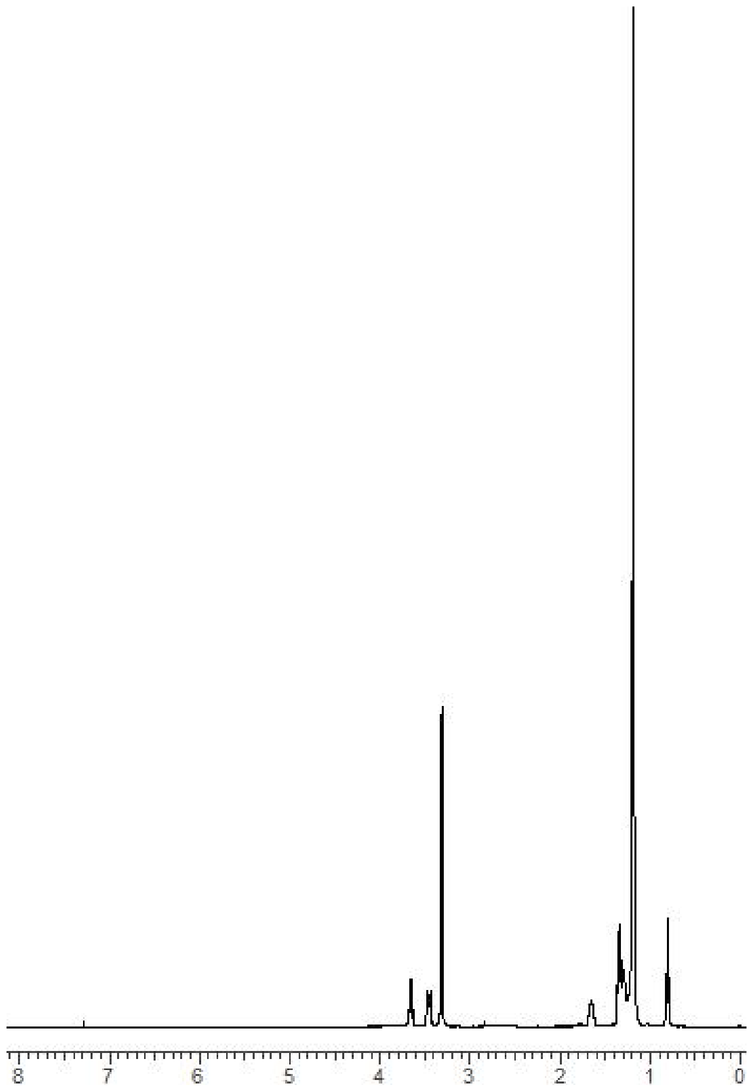

Figure S4. ^{1}H NMR spectrum of $C_{18}DMEAB$ in $CDCl_3$ at 25 °C.

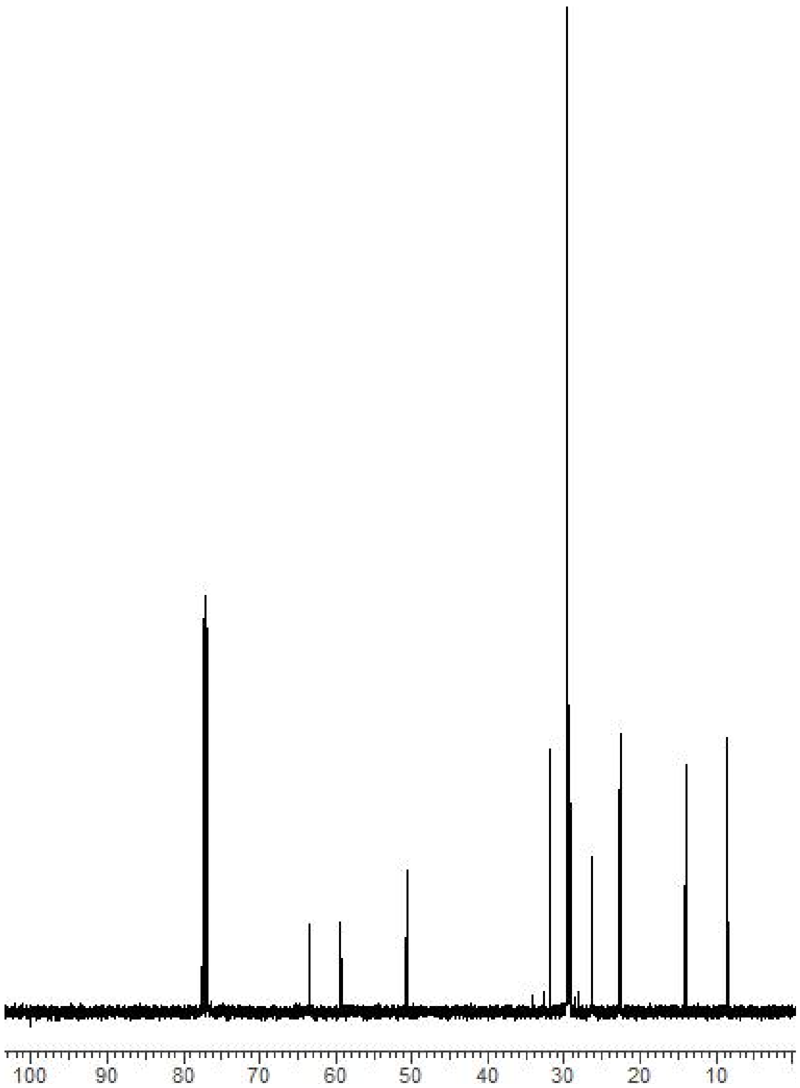

Figure S5. $^{13}C\{^1H\}$ NMR spectrum of $C_{18}DMEAB$ in $CDCl_3$ (77.16 ppm) at 25 °C.

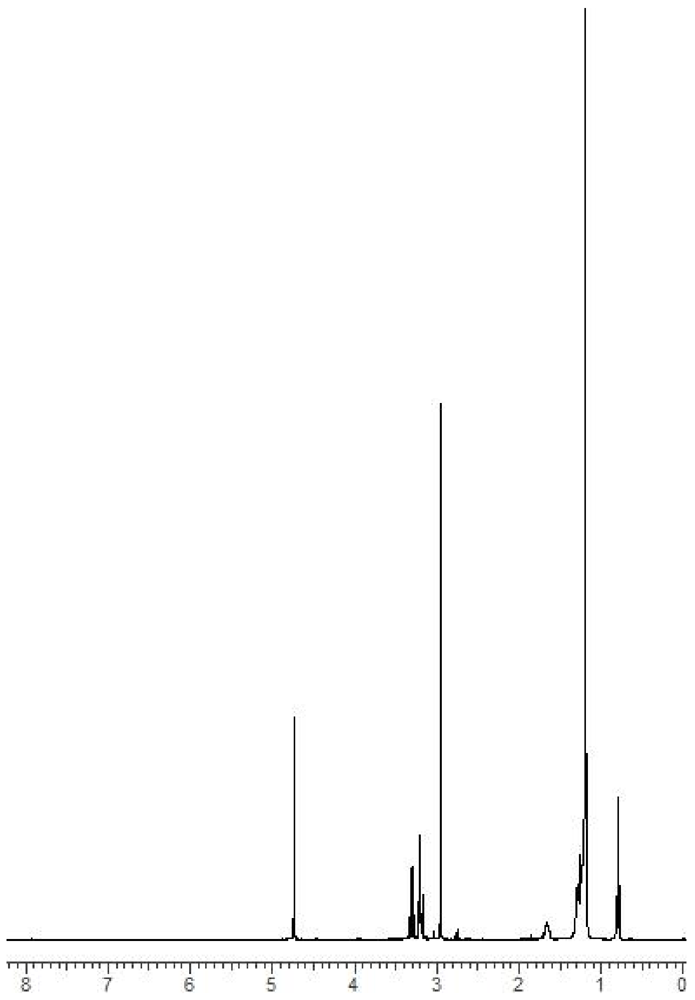

Figure S6. ^{1}H NMR spectrum of C_{18}DEMAB in D-methanol at 25 °C.

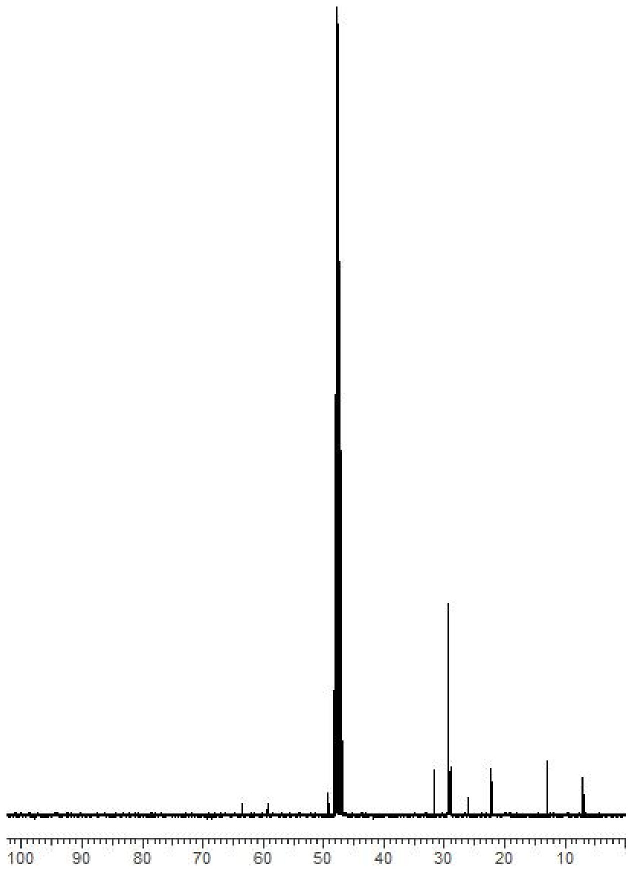

Figure S7. $^{13}C\{^1H\}$ NMR spectrum of $C_{18}DEMAB$ in D-methanol at 25 ℃.

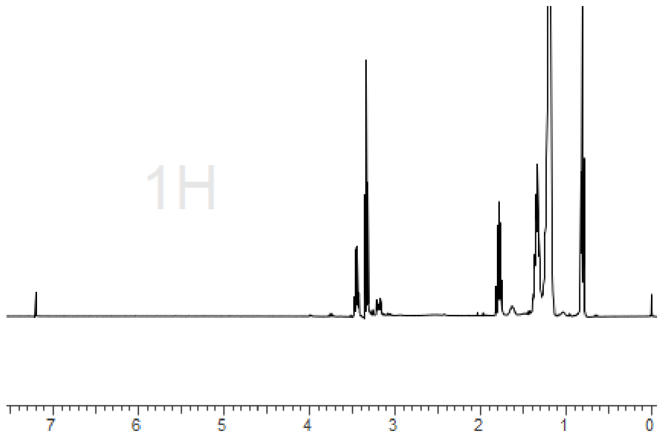

Figure S8. ^1H NMR spectrum of $C_{18}TEAB$ in $CDCl_3$ at 25 ℃.

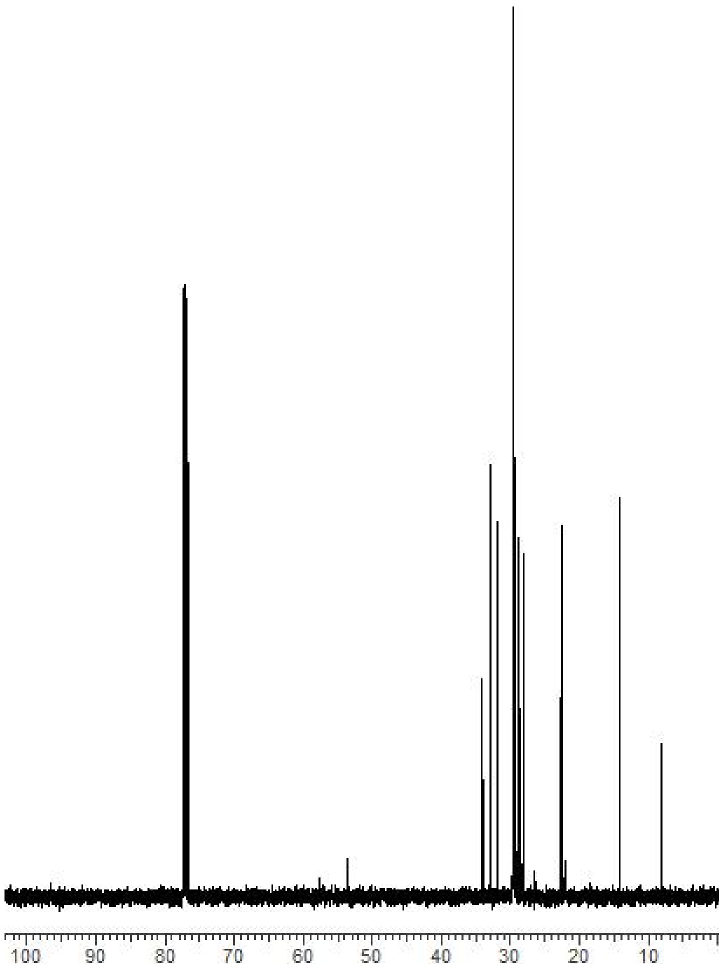

Figure S9. $^{13}C\{^{1}H\}$ NMR of $C_{18}TEAB$ in $CDCl_3$ (77.16 ppm) at 25 °C.

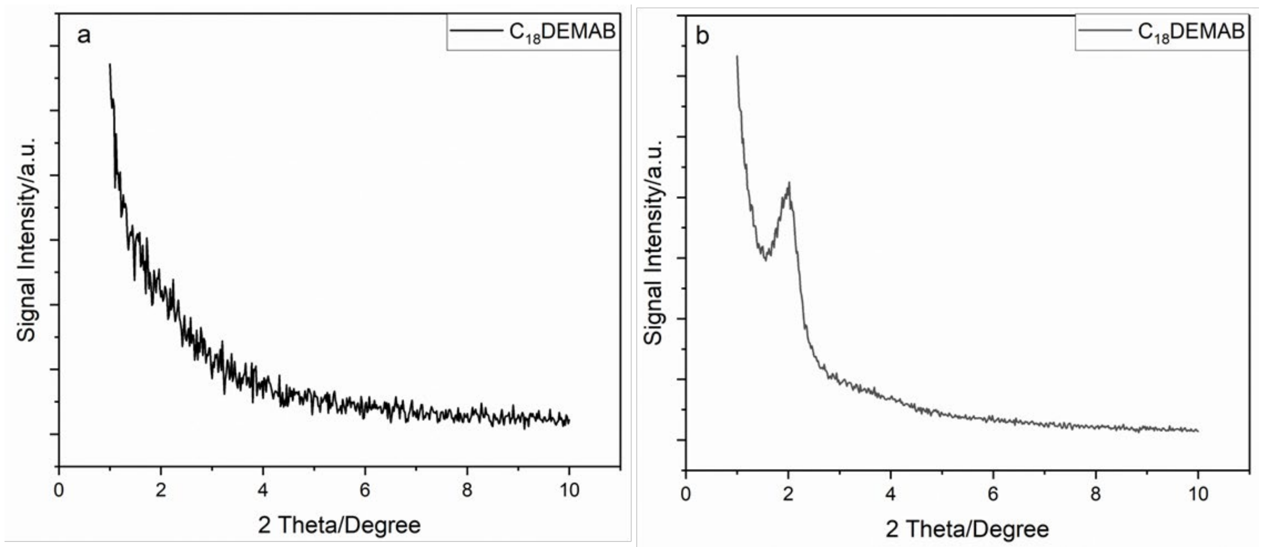

Figure S10 1D in-plane GISAXS patterns of EASA films produced with $C_{18}DEMAB$ (a) before (b) after surfactant removal. The film was deposited at a potential of -1.25 V (vs. Ag/Ag^{+}) for 20 seconds on ITO electrodes.

Table S1. The electrochemical data of a range of mesoporous silica films collected from CV's

Surfactants/Electrode	Redox Probe	I_{pa} (mV)	I_{pc} (mV)	ΔE_p (mV)
Bare ITO	$[Fe(CN)_6]^{3-/4-}$	2.02×10^{-2}	-2.03×10^{-2}	90
C_{18}TAB	$[Fe(CN)_6]^{3-/4-}$	1.35×10^{-2}	-1.26×10^{-2}	148
C_{18}DMEAB	$[Fe(CN)_6]^{3-/4-}$	1.58×10^{-3}	-3.01×10^{-3}	104
C_{18}DEMAB	$[Fe(CN)_6]^{3-/4-}$	N/A	N/A	N/A
C_{18}TEAB	$[Fe(CN)_6]^{3-/4-}$	N/A	N/A	N/A
